# Evaluation of inactivated *Bordetella pertussis* as a delivery system for the immunization of mice with Pneumococcal Surface Antigen A

**DOI:** 10.1371/journal.pone.0228055

**Published:** 2020-01-16

**Authors:** Julia T. Castro, Giuliana S. Oliveira, Melissa A. Nishigasako, Anne-Sophie Debrie, Eliane N. Miyaji, Alessandra Soares-Schanoski, Milena A. Akamatsu, Camille Locht, Paulo L. Ho, Nathalie Mielcarek, Maria Leonor S. Oliveira

**Affiliations:** 1 Laboratório de Bacteriologia, Instituto Butantan, São Paulo, SP, Brazil; 2 Univ. Lille, CNRS, Inserm, CHU Lille, Institut Pasteur de Lille, U1019 –UMR 8204 –CIIL—Center for Infection and Immunity of Lille, Lille, France; 3 Seção de Vacinas Aeróbicas, Divisão Bioindustrial, Instituto Butantan, São Paulo, SP, Brazil; University of South Dakota, UNITED STATES

## Abstract

Pneumococcal Surface Protein A (PspA) has been successfully tested as vaccine candidate against *Streptococcus pneumoniae* infections. Vaccines able to induce PspA-specific antibodies and Th1 cytokines usually provide protection in mice. We have shown that the whole cell pertussis vaccine (wP) or components from acellular pertussis vaccines, such as Pertussis Toxin or Filamentous Hemagglutinin (FHA), are good adjuvants to PspA, suggesting that combined pertussis-PspA vaccines would be interesting strategies against the two infections. Here, we evaluated the potential of wP as a delivery vector to PspA. *Bordetella pertussis* strains producing a PspA from clade 4 (PspA4Pro) fused to the N-terminal region of FHA (Fha44) were constructed and inactivated with formaldehyde for the production of wP^PspA4Pro^. Subcutaneous immunization of mice with wP^PspA4Pro^ induced low levels of anti-PspA4 IgG, even after 3 doses, and did not protect against a lethal pneumococcal challenge. Prime-boost strategies using wP^PspA4Pro^ and PspA4Pro showed that there was no advantage in using the wP^PspA4Pro^ vaccine. Immunization of mice with purified PspA4Pro induced higher levels of antibodies and protection against pneumococcal infection than the prime-boost strategies. Finally, purified Fha44:PspA4Pro induced high levels of anti-PspA4Pro IgG, but no protection, suggesting that the antibodies induced by the fusion protein were not directed to protective epitopes.

## Introduction

Lower respiratory infections are among the most important causes of death globally, affecting more than two million people from all ages in 2016 [1]. *Streptococcus pneumoniae* (pneumococci) is the most frequent etiological agent, contributing with more than 1 million deaths in all ages and around 350 thousand in children under 5 years of age [1]. After almost 20 years of use, pneumococcal conjugate vaccines, composed of polysaccharides from prevalent pneumococcal serotypes conjugated to protein carriers, have greatly contributed to reductions in pneumococcal colonization and invasive diseases around the world [2,3]. However, increase in diseases caused by non-vaccine serotypes were observed in several countries and may affect vaccine efficacy against pneumococcal diseases in different populations [4]. Pneumococcal proteins are alternatives for the development of vaccines with broad-serotype coverage [5]. Pneumococcal Surface Protein A (PspA) is a virulence factor that helps bacteria to escape the immune system by interfering with complement deposition on its surface [6] and with the bactericidal activity of the host apolactoferrin [7].

We have previously shown that the whole cell pertussis vaccine (wP) is a potent adjuvant to PspA, able to enhance the induction of specific antibodies and protection against invasive pneumococcal infection and nasal colonization in animal models [8,9]. Besides the whole bacteria, Pertussis toxin (PT) and Filamentous hemagglutinin (FHA) can also exert adjuvant activity when combined to PspA [10] leading us to propose that combined pertussis- PspA vaccines could be interesting approaches to immunize against infections caused by both *Bordetella pertussis* and pneumococci.

Instituto Butantan, São Paulo, Brazil, produces the wP vaccine that is administered as DTwP (triple Diphtheria, Tetanus, Pertussis vaccine) to Brazilian children since 1980. Here, we have tested the potential of wP as a delivery system for PspA, by the construction of a recombinant *B*. *pertussis* expressing a fusion of PspA4Pro (the N-terminal region of PspA from clade 4 including a proline rich sequence) with the N-terminal fragment of FHA (Fha44:PspA4Pro), using the Brazilian vaccine strain. Mice were immunized with wP^PspA4Pro^ and the induction of anti-PspA antibodies, as well as protection against pneumococcal infection were analyzed. The potential of purified recombinant Fha44:PspA4Pro protein as a vaccine candidate was also tested.

## Materials and methods

### Ethics statement

This study was performed according to the guidelines outlined by the Brazilian National Council for Control of Animal Experimentation (CONCEA). Experimental protocols were approved by the Ethic Committee on Animal Use of the Butantan Institute (CEUAIB) (protocol numbers 1363/15 and 3154200117). Six animals were housed per cage inside a ventilated cabinet under controlled temperature and light cycle (12/12 hours, light/dark cycle) with daily monitoring in a BSL2 animal facility. Food and water were given ad libitum. Monitoring and manipulation was done by trained personnel.

### Bacteria and plasmids construction

*B*. *pertussis* NIH137[11] is the vaccine strain used for the production of wP at Instituto Butantan, São Paulo, Brazil and was used in this work. *B*. *pertussis* 18323 (used in the potency tests for of wP vaccines at Instituto Butantan) was used for the production of *B*. *pertussis* protein lysates. Bacteria were grown on Bordet-Gengou (BG–Difco, New Jersey, USA) agar plates, supplemented with 1% glycerol and 20% defibrinated sheep blood, at 35°C. Nalidixic acid (Nal, 30 μg/mL) or gentamycin (Gm,10 μg/mL) were added to the BG-blood agar plates when required. *Escherichia coli* DH5α SM-10 λ Pir or BL21 (DE3) star pLysS were grown in Luria Bertani (LB—Difco), supplemented with 10μg/mL Gm or 100 μg/mL ampicillin for the selection of plasmid-containing clones. *S*. *pneumoniae* ATCC6303 (serotype 3) was grown on Triptic soy agar containing 5% defibrinated sheep blood (Laborclin, SP, Brazil) at 37°C. Stocks for challenge were prepared in liquid Todd-Hewitt media (Difco) supplemented with 0.5% yeast extract (THY). Bacteria were grown until exponential phase (OD600_nm_ 0.4), centrifuged, and suspended in 1:10 of the initial volume in THY containing 40% glycerol. Stocks were maintained at -80°C and quantified by plating on blood agar. The plasmid pXR1Fha44 [12], which confers resistance to Gm, was used for the expression of PspA4 in *B*. *pertussis* NIH137. The sequence coding for the N-terminal region of PspA from clade 4, containing a proline block (PspA4Pro) was amplified by PCR from the pAE-PspA4Pro plasmid [13] using the oligonucleotides 5’ ACGCGTGTAAGAGCAGAAGAAGCC 3’ and 5’ CATATGTGGTTTTGGTGCTGGAGC 3’.The fragment was inserted between the MluI and NdeI restriction sites of pXR1Fha44, yielding a plasmid that contains a hybrid gene coding for the N-terminal region of filamentous hemagglutinin (Fha44) fused to PspA4Pro. For the expression of the recombinant protein in *E*. *coli*, a PCR for the amplification of the fragment coding for the Fha44:PspA4Pro excluding the *fha* signal peptide was performed using the oligonucleotides 5’ GGATCCCAAGGTCTGGTTCCGCAG 3’ and 5’ AAGCTTCGAATTCCTAGGTACCTTATGGTTTTGGT 3’. The sequence was inserted between the BamHI and the HindIII restriction sites in the pAE vector [14], yielding a plasmid that codes for Fha44:PspA4Pro fused to a histidine tag.

### Expression of FHA44:PspA4Pro in *B*. *pertussis* and *E*. *coli*

Conjugation of *B*. *pertussis* NIH137 and *E*. *coli* SM-10 λ pir was performed for the generation of *B*. *pertussis* clones producing Fha44:PspA4Pro (Bp^PspA4Pro^). Briefly, *B*. *pertussis* grown on BG-blood agar was harvested and plated onto a fresh BG-blood agar plate. One colony of SM-10 λ pir transformed with the pXR1Fha44:PspA4Pro plasmid was mixed with *B*. *pertussis* on the plate and incubated at 35°C for 6h. Bacteria were then collected and plated onto BG-blood agar containing Gm and Nal for the selection of recombinant *B*. *pertussis* and elimination of *E*. *coli*. Clones able to grow on Gm were selected once again on Nal to confirm proper selection. Stocks of the recombinant clones were maintained at -80°C and expression was evaluated by western-blot of protein extracts using anti-PspA4 or anti-FHA polyclonal antisera (produced at the laboratory) and Amersham ECL Prime detection reagent (GE Healthcare).

Purified recombinant Fha44:PspA4Pro proteins were obtained from *E*. *coli* BL21 Star (DE3) pLysS transformed with the pAE-Fha44:PspA4Pro plasmid as follows. Recombinant bacteria were grown in LB-amp until OD_600nm_ = 0.5 and expression of the protein was induced by the addition of 1.2mM IPTG for 3h. Protein expression was confirmed by SDS-PAGE. Bacteria were harvested by centrifugation and lysates were prepared in a PANDA homogenizer (GEA Niro Soavi, Parma, Italy). The Fha44:PspA4Pro protein was detected in inclusion bodies that were dissolved in 50mM Tris (pH 8.0), 150mM NaCl, 5 mM imidazole, 8M urea. Purification of the recombinant protein was performed through affinity chromatography using 5mL His Trap columns (GE Healthcare, Illinois, USA) in the AKTA Prime equipment (GE Healthcare). After the adsorption of the protein to the column, washing steps with increasing concentrations of imidazole were performed and protein was eluted in 250mM imidazole. 8 M urea was maintained during the entire process and the purified Fha44:PspA4Pro was also stored in 8 M urea, since efforts to remove it by dilution or dialysis resulted in the precipitation of the protein. Purified PspA4Pro [15] was kindly provided by Dr. Viviane Maimoni Gonçalves, Instituto Butantan, São Paulo, Brazil. SDS-PAGE and Western blots images were produced using the KodaK Gel Logic 200 equipment with the Carestream software except for S2 Fig. that was produced using the Amersham Image 680 equipment.

### Production of wP^PspAPro^ vaccines and immunization of mice

*B*. *pertussis* producing Fha44:PspA4Pro was grown on BG-blood agar at 35°C. Bacteria were suspended in non-pyrogenic PBS (Gibco) to an OD_600nm_ = 1.8 and washed once in PBS. Pellets were suspended in 1/10 of the initial volume of PBS and aliquots were used for quantification by plating on BG-agar plates containing Gm. Samples were centrifuged, suspended in the same volume of PBS containing 0.2% formaldehyde and incubated at 35°C for 24h under agitation. Bacteria were then washed once in PBS and suspended in the same volume of PBS. This protocol was based on that used for the production of wP at Instituto Butantan. Non-recombinant *B*. *pertussis* NIH137 was subjected to the same protocol as a control. wP and wP^PspA4Pro^ vaccines were maintained at 4°C until use.

Female SPF BALB/c mice were produced by the animal facility from the Medical School, University of São Paulo. Animals (6 per group) were supplied with food and water *ad libitum*. Mice were immunized through the subcutaneous route with the equivalent of 1x10^9^ CFU of *B*. *pertussis*, in a total volume of 100μL, with booster doses given at 15-days intervals, up to three doses. In prime-boost protocols, mice received the same dose of bacteria,1μg of PspA4Pro or saline through the subcutaneous route, followed by two boosters with saline or PspA4Pro. For the experiments with purified recombinant Fha44:PspA4Pro, mice were immunized twice subcutaneously at a 15-day interval with 1μg of protein. FHA (Sigma-Aldrich, Missouri, USA) and PspA4Pro were used as controls. Blood was collected 14 days after each immunization through the retroorbital plexus, under local anesthesia with 5% proxymetacaine chloride eye drops (Alcon, Texas, USA). Blood was incubated at 37°C for 30 min, following by incubation at 4°C for 10 min. Samples were centrifuged at 800 x *g* for 10 min for the collection of sera. Induction of IgG, IgG1 and IgG2a was evaluated by ELISA using plates coated with 1μg/mL of PspA4Pro or protein lysates from *B*. *pertussis* 18323. Sera collected from immunized mice were tested in serial dilutions and Horseradish Peroxidase anti-mice IgG, IgG1 or IgG2a (Southern Biotech, Alabama, USA) were used as secondary antibodies. Reactions were developed using o-Phenylenediamine dihydrochloride as substrate and the absorbances were measured at 492nm in a spectrophotometer multiskan EX (Thermo Fisher Scientific, Massachusetts, USA). Antibody titers were defined as the reciprocal of the dilution that produced an absorbance of 0.1.

### Antibody binding and complement deposition to pneumococcal surface

*S*. *pneumoniae* ATCC6303 was grown on blood agar plates for 18h. Bacteria were diluted in THY, grown until OD_600 nm =_ 0.4–0.5 (~10^8^ CFU/mL) and harvested by centrifugation at 3,200 x *g* for 10 min. Bacteria were then washed, suspended in PBS and incubated with 5% (V/V) of sera from immunized mice (pooled sera from each experimental group) during 30 min on ice. Samples were then washed once with PBS before incubation for 30 min with fluorescein isothiocyanate (FITC)-conjugated goat anti-mouse IgG (MP Biomedicals, California, USA), diluted 1:100 in PBS. For complement deposition assays, sera were previously inactivated at 56°C for 30 min and incubated with bacteria at a concentration of 10 or 20%, for 30 min on ice. Samples were washed once with PBS and incubated with 10% of normal mouse serum as source of complement, in Gelatin Veronal buffer (Sigma Aldrich), at 37 ºC for 30 min. After washing, samples were incubated with FITC-conjugated anti-mouse C3 IgG (MP Biomedicals) in PBS, for 30 min on ice. In both experiments, samples were fixed with 200 μL of cytofix (BD Biosciences, California, USA) after two washing steps. Flow cytometry analysis was conducted using FACS Canto II (BD Biosciences), and 15,000 gated events were recorded. Fluorescence was analyzed by histograms using the Flow Jo 10.1 software and medians of the curves were used to compare the groups.

### Intranasal Pneumococcal challenge

Immunized mice were challenged with *S*. *pneumoniae* ATCC6303 by the intranasal inoculation of 3X10^5^ bacteria in 50μL saline, 21 days after the last immunization. Challenge was performed under anesthesia, using 100 mg/Kg of ketamine chloride (Ceva, São Paulo, Brazil) and 20mg/Kg of xylazine chloride (Ceva) intraperitoneally and survival was followed for up to 15 days. Animals were monitored twice daily after challenge and lethargic animals with reduced ability to move were euthanized immediately through the ip route with a lethal dose of a xylazine/ketamine solution (60 mg/Kg of xylazine and 300 mg/Kg of ketamine). No animals died before meeting criteria for euthanasia.

### Statistical analyses

Differences in antibody concentrations were analyzed by One-way ANOVA followed by Tukey´s post-test for the comparisons between two groups. Survival was analyzed by the Log-rank survival curve using the Mantel-Cox test for the comparison between groups. Statistical analyses were performed using Prism 6.0 software and *P*≤0.05 was considered significantly different.

## Results

### Recombinant *B*. *pertussis* produces Fha44:PspA4Pro

**Fig 1:**
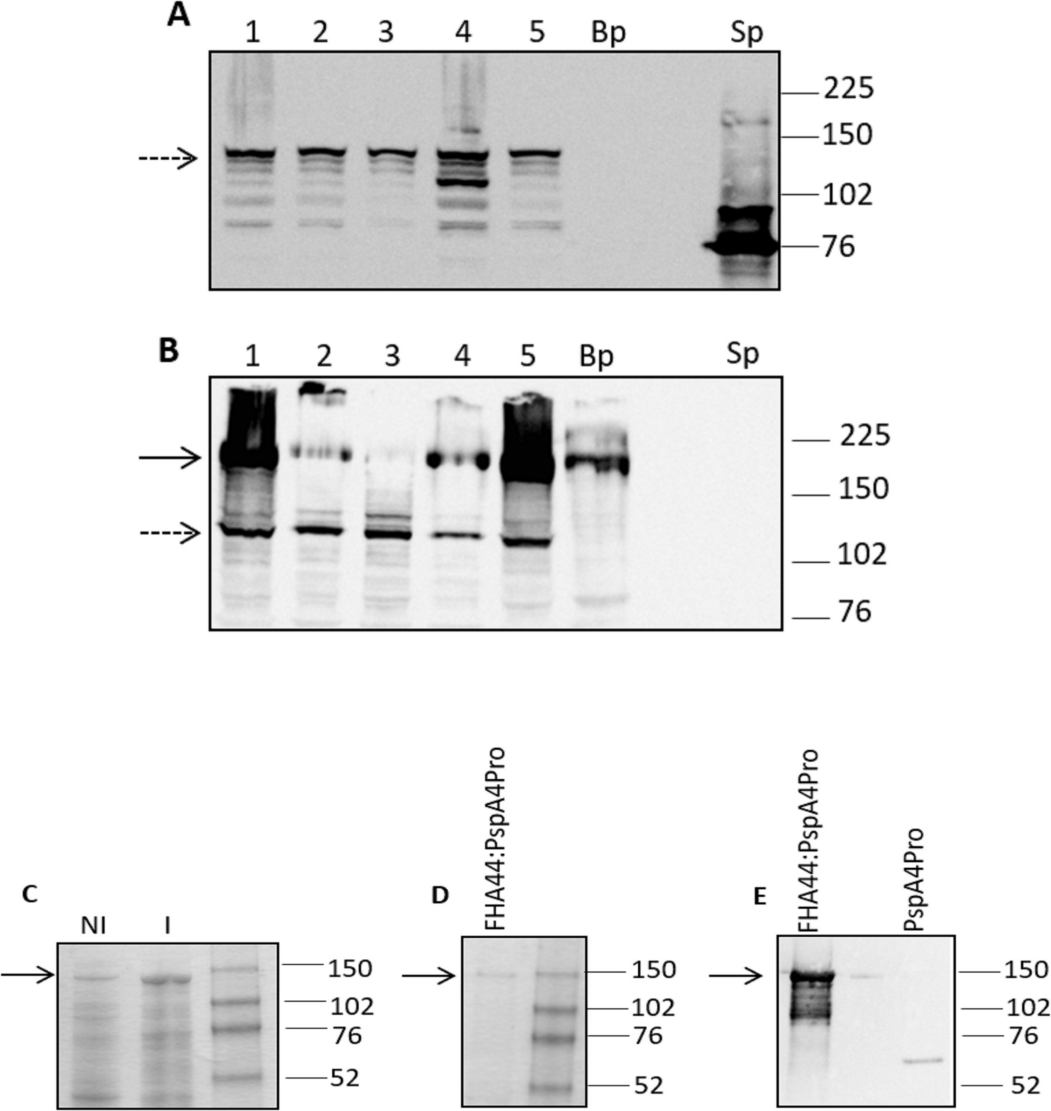
Expression of FHA44:PspA4Pro. (A and B) Lysates of 5 recombinant *B*. *pertussis* clones or non-recombinant *B*. *pertussis* (Bp) were evaluated by western-blot using anti-PspA **(A)** or anti-FHA antisera **(B)**. The 255/00 pneumococcal strain lysate was used as a PspA4 control (Sp). (C and D) FHA44:PspA4Pro was also expressed in *E*. *coli*, NI, lysate from a representative clone in non-induced condition; I, lysate from the same clone after induction with IPTG **(C)** and purified by affinity chromatography **(D)**. Identification of the purified recombinant protein was performed by western-blot using anti-PspA4 antisera (E). In (C), 15 μg of total protein were applied to the gel; In D and E, 0.5 μg of purified protein was applied to the gel. In all figures the GE Healthcare rainbow full range molecular weight marker was used.

*B*. *pertussis* NIH137 was conjugated with *E*. *coli* SM-10 λ Pir (pXR1Fha44:PspA4Pro) to generate clones producing Fha44:PspA4Pro (Bp^PspA4Pro^). In three independent experiments, several clones were selected with expression of the protein of interest. Western blot analysis using anti-PspA4 antisera revealed a band of 130kDa, the expected size for Fha44:PspA4Pro, in 5 representative clones of Bp^PspA4Pro^ and no reactivity with extracts from non-recombinant *B*. *pertussis* NIH137 (Bp) (Fig 1A). In addition, lower-M_*r*_ bands reacting with the anti-PspA4 antisera were observed, suggesting partial degradation. The same clones were also examined using anti-FHA antisera. As shown in Fig 1B, the anti-FHA antisera revealed a band of around 220 kDa corresponding to full-length FHA produced by BP^PspA4Pro^ as well as by Bp, albeit with variable intensity. The 130kDa band, corresponding to Fha44:PspA4Pro, was only observed on Bp^PspA4Pro^. In order to evaluate whether the fusion with Fha44 had driven PspA4Pro to the surface of the bacteria, flow cytometry on two Bp^PspA4Pro^ clones and Bp NIH137 was performed, using anti-FHA and anti-PspA4 antisera. S1 Fig shows positive reactivity of all bacteria with anti-FHA antibodies and low but positive reactivity of the Bp^PspA4Pro^ clones with the anti-PspA4 antisera, indicating that at least part of the Fha44:PspA4Pro protein is exposed on the surface of the recombinant bacteria.

Using the pAE vector, expression of Fha44:PspA4Pro was also observed in recombinant *E*. *coli* after induction with IPTG (Fig 1C). The purified protein obtained by Ni^2+^- affinity chromatography (Fig 1D) was also detected by the anti-PspA4 antisera (Fig 1E).

### wP^PspA4Pro^ vaccines induce low levels of anti-PspA4 antibodies and are not protective against pneumococcal infection

**Fig 2:**
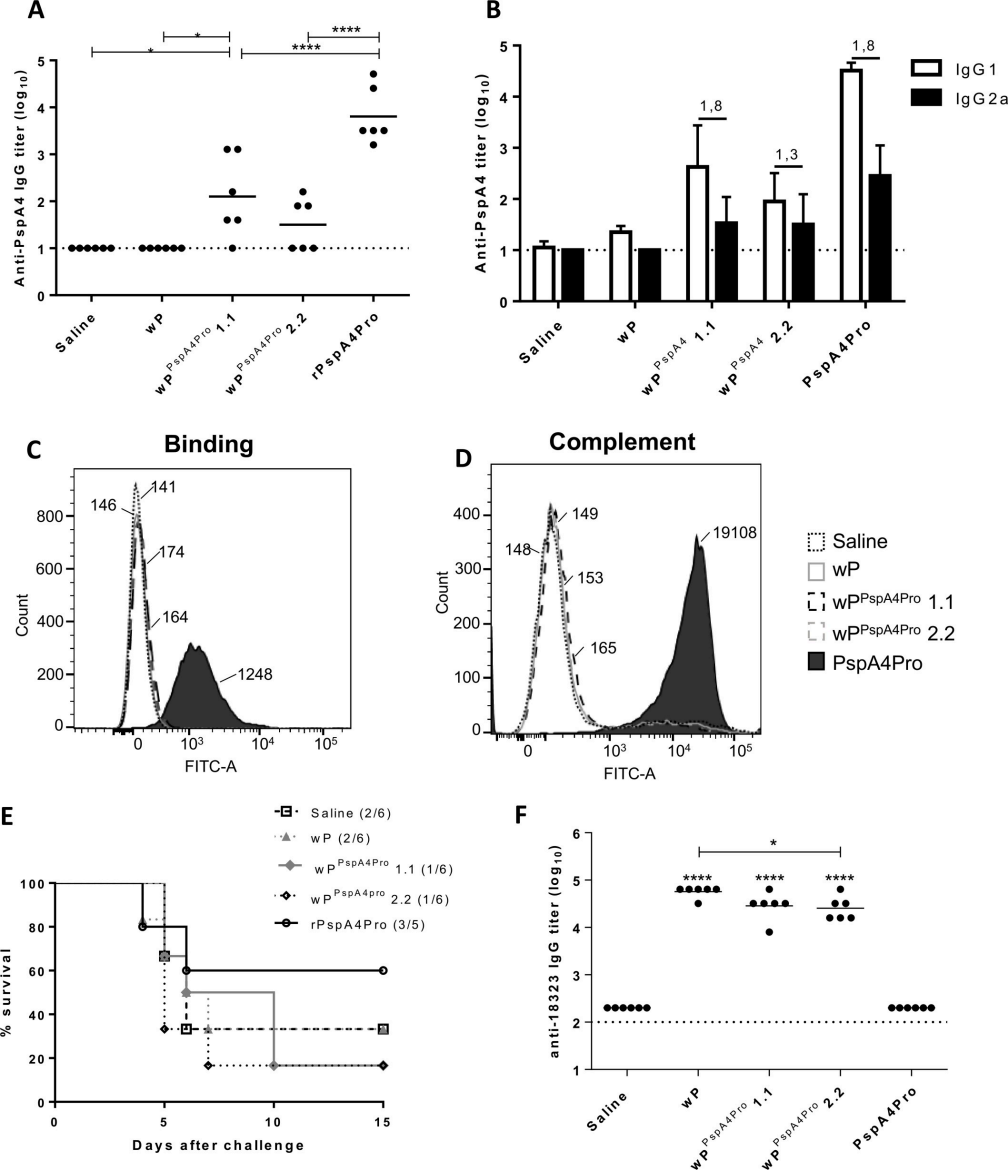
wP^PspA4Pro^ induces low levels of anti-PspA4 IgG. BALB/c mice were immunized with three doses of wP^PspA4Pro^, wP or PspA4Pro. (A, B and F) Anti-PspA4Pro IgG (A), IgG1 and IgG2a (B) or anti-*B*. *pertussis* 18323 protein lysates (F) were measured by ELISA. (A and E), circles represent individual data and lines represent the means for each group. (B), bars represent the means for each group with the standard deviations; numbers above the bars indicate the IgG1:IgG2a ratios. Titers in control groups were below the limit of detection and, therefore IgG1:IgG2a ratios were not calculated for them. (C and D) Pools of sera from each group of mice were evaluated for the capacity to bind (C) or to induce complement deposition (D) on the surface of *S*. *pneumoniae* ATCC6303 by flow cytometry. Numbers indicate the medians of fluorescence intensity for each curve. (E) Intranasal challenge with *S*. *pneumoniae* ATCC6303 was performed in immunized mice and survival was followed for 15 days. *P<0.05; ****P<0.0001, One-way ANOVA with Tukey post-test or Log-Rank survival curve with Mantel-Cox test (E).

Selected clones were used for the production of inactivated wP^PspA4Pro^ vaccines. Mice received three doses of the inactivated vaccines wp^PspA4Pro^ or wP (equivalent of 1x10^9^ CFU of *B*. *pertussis* per dose). An estimative made by comparative western-blot analysis, based on a curve with purified Fha44:PspA4Pro, indicated that around 10ng of the protein was present in each dose (S2 Fig). Purified PspA4Pro was used as a positive control in the immunization experiment. Even after three doses, only low levels of anti-PspA4Pro IgG and highly variable responses among the animals were observed in the groups of mice immunized with wP^PspA4Pro^, produced from two different clones (Fig 2A). In contrast, high levels of anti-PspA4Pro IgG were observed in mice immunized with three doses of the recombinant protein. Characterization of the IgG subtypes revealed balanced IgG1:IgG2a ratios in the immunized groups, with no significant differences observed between animal vaccinated with wP^PspA4Pro^ or PspA4Pro (Fig 2B).

Reactivity of the antisera with native PspA was tested *in vitro* by binding assays to the surface of *S*. *pneumoniae* ATCC6303. Only the sera from mice immunized with PspA4Pro were able to recognize the protein on the pneumococcal surface (median of fluorescence intensity of 1248, Fig 2C). Sera from mice immunized with wP^PspA4Pro^ vaccines produced from two different clones, showed reactivity at the same levels of the sera obtained from mice immunized with saline or the wP vaccine (with medians of fluorescence intensity below 200), probably reflecting the low levels of anti-PspA4Pro IgG induced by these vaccines. In addition, only the sera from mice immunized with PspA4Pro induced significant deposition of complement C3 on pneumococcal surface (median of fluorescence intensity of 19108, Fig 2D).

Immunized mice were challenged with *S*. *pneumoniae* ATCC6303 by the intranasal route and only PspA4Pro was able to induce 60% survival of mice, a level usually observed by our group when the recombinant protein is administered to mice without any adjuvant [8]. No improvement in survival of mice immunized with the wP^PspA4Pro^ vaccines was observed when compared with the controls immunized with wP or saline (Fig 2E).

We then analyzed the induction of antibodies against *B*. *pertussis* antigens by the wP^PspA4Pro^ vaccine. Sera collected from mice after the second dose showed high levels of IgG reacting with total protein extracts from *B*. *pertussis* 18323. No differences in the levels of antibodies were observed when comparing wP with wP^PspA4Pro^ vaccines (Fig 2F). As expected, purified recombinant PspA4Pro did not induce any anti-*B*. *pertussis* antibodies. Thus, expression of PspA4Pro did not affect the response to *B*. *pertussis* antigens and the vaccine formulations were immunogenic in mice.

### Prime-boost protocols using wP^PspAPro^ and PspA4Pro do not protect mice against pneumococcal respiratory infection

**Fig 3:**
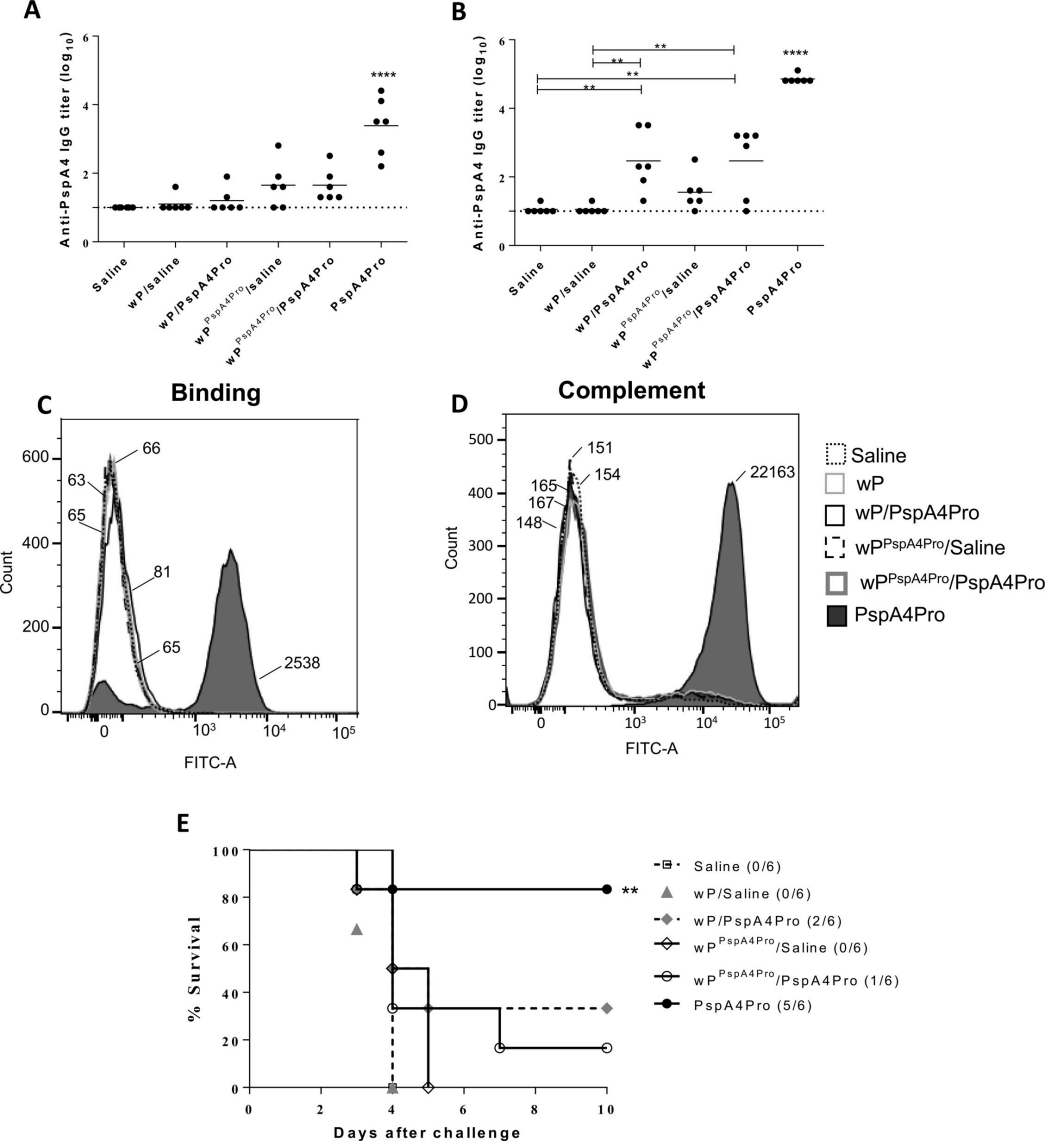
Prime-boost protocol with wP^PspA4Pro^ and PspA4Pro does not improve protection against pneumococcal infection. (A and B) BALB/c mice were immunized with one dose of wP or wP^PspA4Pro^ followed by one (A) or two doses (B) of saline or PspA4Pro. Control mice received only PspA4Pro. Anti-PspA4Pro IgG were measured by ELISA. Circles represent individual data and lines represent the means for each group. (C and D) Pools of sera from each group of mice were evaluated for the capacity to bind (C) or to induce complement deposition (D) on the surface of *S*. *pneumoniae* ATCC6303 by flow cytometry. Numbers indicate the medians of fluorescence intensity for each curve. (E) Intranasal challenge with *S*. *pneumoniae* ATCC6303 was performed in immunized mice and survival was followed for 15 days. **P<0.01; ****P<0.0001, One-way ANOVA with Tukey post-test (A and B) or Log-Rank survival curve with Mantel-Cox test (E).

Since immunization with wP^PspA4Pro^ vaccines did not induce high levels of antibodies nor did they protect mice against invasive pneumococcal infection, we tested prime-boost protocols using recombinant PspA4Pro as boost, to evaluate possible benefits on the modulation of the immune system by the inactivated vaccine. Mice received wP^PspA4Pro^ as the priming vaccine and saline or PspA4Pro in the booster vaccine. As controls, mice were primed with wP or PspA4Pro. As shown in Fig 3A, the prime-boost regimen using wP^PspA4Pro^ followed by one dose of PspA4Pro induced low levels of anti-PspA4Pro antibodies, that were comparable to the levels observed in mice immunized with wP^PspA4Pro^ followed by saline or wP followed by PspA4Pro. In contrast, high levels of anti-PspA4Pro IgG were observed in mice immunized with two doses of the recombinant protein. A third dose was given to the animals and, the highest levels of anti-PspA4Pro IgG were again induced in animals immunized with three doses of the recombinant protein (Fig 3B). No differences between the groups of mice primed with wP or wP^PspA4Pro^ were observed, indicating no benefits for the use of wP^PspA4Pro^. Sera collected after the third dose were tested for binding to the surface of *S*. *pneumoniae* ATCC6303. Binding of IgG was only detected in sera from mice immunized with three doses of recombinant PspA4Pro (Fig 3C) and this was the only sera that induced the deposition of complement C3 on bacterial surface (Fig 3D). In addition, this was the only group of mice that were significantly protected against the invasive challenge with *S*. *pneumoniae* ATCC6303 (Fig 3E).

### Fha44:PspA4Pro induces similar levels of anti-PspA4Pro IgG but no protection against pneumococcal infection

**Fig 4:**
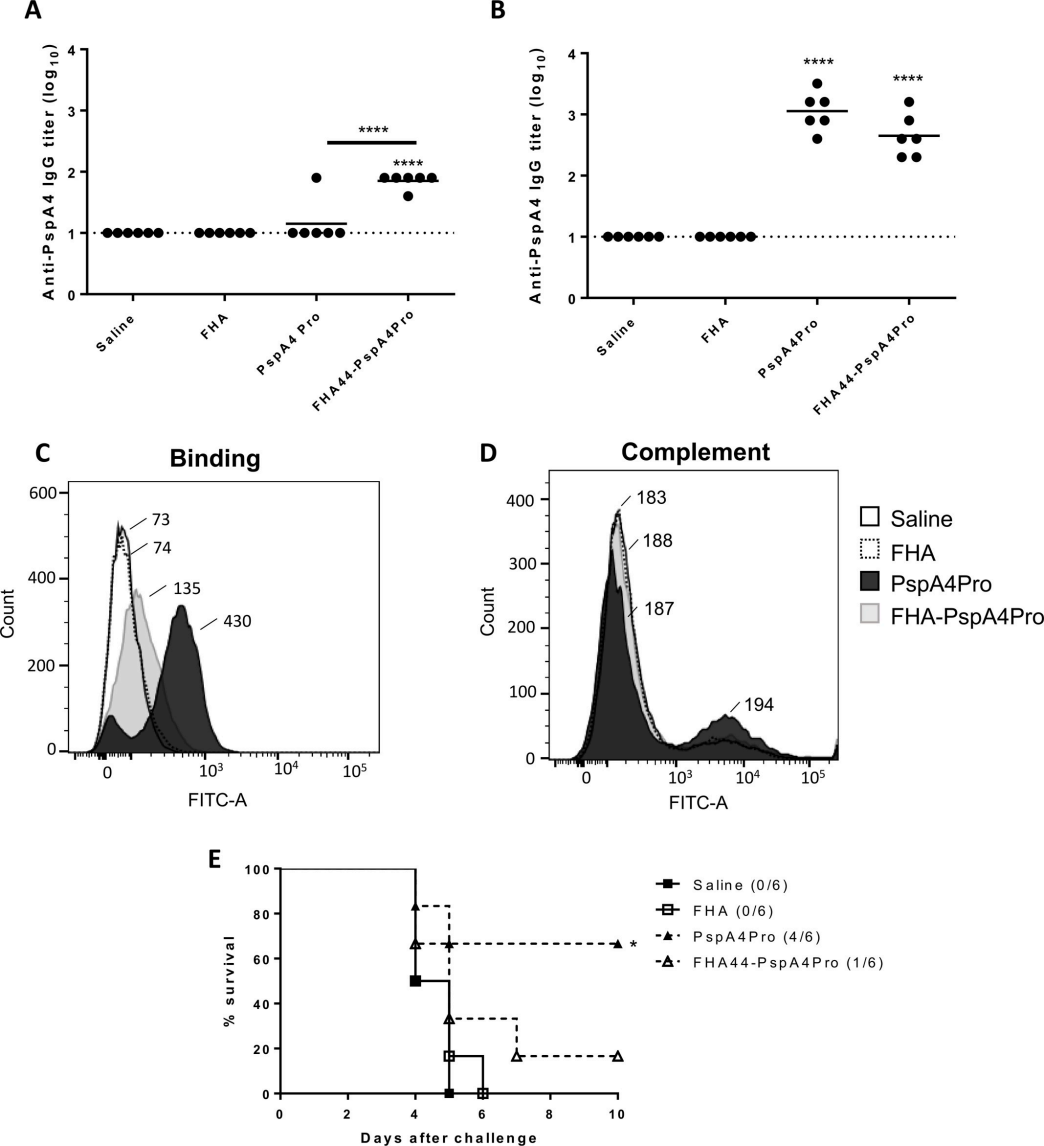
FHA44:PspA4Pro induces anti-PspA4Pro IgG but no protection against pneumococcal infection. (A and B) BALB/c mice were immunized with one (A) or two (B) doses of FHA44:PspA4. FHA or PspA4Pro were used as negative or positive controls, respectively. Anti-PspA4Pro IgG were measured by ELISA. Circles represent individual data and lines represent the means for each group. Pools of sera from each group of mice were evaluated for the capacity to bind (C) or to induce complement deposition (D) on the surface of *S*. *pneumoniae* ATCC6303 by flow cytometry. Numbers indicate the medians of fluorescence intensity for each curve. (E) Intranasal challenge with *S*. *pneumoniae* ATCC6303 was performed in immunized mice and survival was followed for 15 days. *P<0.05; ****P<0.0001, One-way ANOVA with Tukey post-test (A and B) or Log-Rank survival curve with Mantel-Cox test (E).

Adjuvant properties have been described for different *B*. *pertussis* antigens, including PT and FHA. We therefore tested purified Fha44:PspA4Pro as a vaccine candidate against pneumococcal infection. A single dose of Fha44:PspA4Pro indeed induced higher levels of anti-PspA4Pro IgG compared with PspA4Pro (Fig 4A). However, after one dose neither protein induced antibodies at levels that usually afford protection against pneumococcal invasive infection in mice. After the second dose, the superior response of Fha44:PspA4Pro over PspA4Pro was no longer observed (Fig 4B). Both proteins induced high levels of anti-PspA4Pro IgG, with no significant differences between them. However, IgG induced by PspA4Pro was able to bind more effectively to the *S*. *pneumoniae* ATCC6303 surface, compared to the IgG induced by Fha44:PspA4Pro (Fig 4C), although low levels of complement deposition was observed (Fig 4D), probably due to the relative low binding capacity of the sera after only two doses of PspA4Pro. This activity reflected in lower protective capacity of Fha44:PspA4Pro against the pneumococcal infection when compared with PspA4Pro (Fig 4E).

## Discussion

Different studies have shown that PspA can induce protective immunity to pneumococcal infection in animal models. The presence of high levels of anti-PspA antibodies allows efficient deposition of complement onto the pneumococcal surface, leading to opsonophagocytosis and clearance of bacteria [6]. Antibodies elicited by the immunization of humans with PspA were able to protect mice against invasive pneumococcal infection in passive immunization experiments [16]. Moreover, the induction of Th1 responses, with IgG2a against PspA, was related to optimal immunity against lethal infection and positively correlated with protection against pneumococcal nasal colonization in mice, with higher levels of anti-PspA IgG2a present in mice with lower bacterial numbers [17,18]. Such responses can be induced by bacterial delivery systems, such as live attenuated *Salmonella* [19] and lactic acid bacteria [20,21].

*B*. *pertussis* has also proved to be an effective live delivery vector for heterologous antigens. Virulent or attenuated *B*. *pertussis* strains expressing different antigens have been tested as vaccines against different diseases. Nasal immunization of mice with *B*. *pertussis* expressing the *Schistosoma mansoni* glutathione S-transferase (Sm28GST) fused to FHA induced anti-Sm28GST mucosal and systemic antibodies and protection against parasitic infection [22,23]. Similarly, the *Neisseria meningitidis* Transferin-Binding Protein B (TbpB) was expressed in *B*. *pertussis* fused to the N-terminal domain of FHA. Anti-TbpB antibodies, able to induce complement-mediated killing of *N*. *menigitidis*, were induced after single nasal immunization of mice [24]. Live vaccines based on the attenuated *B*. *pertussis* strain BPZE1 were also developed against influenza A virus, by the expression of the M2e epitope fused to FHA. Induction of immune responses against M2e and reduction in viral load after a challenge with influenza virus H1N1/PR8 were observed in mice immunized through the nasal route [12,25]. However, vaccination of mice with BPZE1 was shown to confer non-specific protection against influenza A virus and the expression of M2e in the recombinant strain did not improve this effect [25].

Our group has been working on the use of the adjuvant properties of wP for the formulation of PspA-based vaccines. Such combined formulations (wP plus recombinant purified PspA) have proved to be effective when inoculated through the nasal and through the subcutaneous routes in mice [8,9].

In the present work, we examined whether a recombinant inactivated pertussis vaccine would also be an effective system to induce immune responses against PspA. Our choice of this approach was due to the fact that inactivated wP vaccines are used in many countries, including Brazil. If effective, this could be a new proposal for DTwP vaccines, with additional protection against pneumococci.

The recombinant NIH137 vaccine strain was able to express PspA4Pro fused to Fha44, as detected by western-blot. Anti-PspA4 antisera also reacted with intact Bp^PspA4Pro^, indicating that the fusion with Fha44 directed PspA4Pro to the surface of *B*. *pertussis*. However, immunization of mice with wP^PspA4Pro^, produced from various clones, did not induce high levels of anti-PspA4Pro antibodies, nor did it confer protection against lethal pneumococcal infection. Several factors may have contributed to the failure to induce robust immune responses against PspA4Pro. The amount of wP and wP^PapA4Pro^ given to mice was based on the potency tests performed for the approval of wP batches for the formulation of DTwP at Butantan Institute. However, our estimates indicated that the amount of Fha44:PspA4Pro in this formulation was very low. Nevertheless, even after three doses, the induction of protective immunity against pneumococcal infection was not observed. This was an important limitation for the use of this system.

The use of formaldehyde to produce the inactivated vaccines was based on the protocol for the wP production by Butantan Institute. This may have affected the production of antibodies against PspA4 epitopes that are important for protection. The failure to observe antibody binding to pneumococcal surface in the sera from mice immunized with wP^PspA4Pro^ also supports the hypothesis that reactivity with the native protein was low.

The combination of FHA with heterologous antigens for the immunization of mice has been shown to increase the induction of antibodies, supporting its adjuvant properties through the nasal, oral or subcutaneous routes [10,26]. The Fha44 fragment, which contains the heparin-binding domain of FHA was proposed as an alternative to the full-length protein in pertussis vaccines [27]. Immunization of mice with Fha44 induces anti-FHA antibodies and protective immunity against *B*. *pertussis* infection in mice. Since it is a smaller and more soluble protein compared to FHA, its purification from a recombinant *B*. *pertussis* strain would have advantages over the full-length antigen. Considering these characteristics and the wP^PspA4Pro^ vaccines evaluated in this work, which contain PspA4Pro fused to Fha44, we tested the purified recombinant fusion protein produced in an *E*. *coli* expression system. However, fusion of PspA4Pro with Fha44 did not improve the induction of anti-PspA antibodies able to protect against pneumococcal infection. Despite the detection of anti-PspA4Pro IgG in sera from mice immunized with Fha44:PspA4Pro by ELISA, binding to native PspA on the pneumococcal surface was less effective compared with sera from mice immunized with PspA4Pro. Moreover, protection of mice immunized with Fha44:PspA4Pro was not observed. These results suggest that important epitopes were not being recognized by sera from mice immunized with Fha44:PspA4Pro. The failure to produce soluble Fha44:PspA4Pro protein in our expression system may have contributed to these results.

Based on the results presented here and the previous results from our group, combination of wP with PspA is a better strategy for a combined pertussis-pneumococcal vaccine than inactivated vaccines produced from recombinant Bp^PspA^ clones. However, the use of live attenuated *B*. *pertussis* expressing PspA against pneumococcal infections remains to be tested. An important difference between the use of recombinant live *B*. *pertussis* vaccines and wP^PspA4Pro^ vaccines, is the fact that the former are able to colonize the respiratory tract of mice for several days after intranasal inoculation, and, therefore, the availability of the heterologous antigens may be prolonged when compared to vaccination with inactivated vaccines.

## Supporting information

S1 Fig Expression of FHA44:PspA4Pro on the surface of *B*. *pertussis*. Expression of FHA and FHA44:PspA4Pro on the surface of Bp137 and two clones of Bp^PspA4Pro^ (1.1 and 2.2) were tested using anti-FHA (A) or anti-PspA4 (B) antisera, by flow cytometry. Bp^PspA4Pro^1.1 without primary sera was used as control. Numbers indicate the medians of fluorescence intensity for each curve.
(TIF)

S2 Fig Estimative of the amount Fha44:PspA4Pro in one dose of wP^PspA4Pro^. Lysates from Bp137 (Bp) and two clones of Bp^PspA4Pro^ (1.1 and 2.2) corresponding to one dose of wP or wP^pspAPro^ vaccines were compared to a curve established with recombinant Fha44:PspA4. Samples were evaluated by western-blot using anti-PspA4 and bands were analyzed using the Amersham Imager 680 analysis software. MW: GE Healthcare rainbow full range molecular weight marker.
(TIF)

S3 Fig Raw image for the production of Fig 1A and 1B. Images were captured with the Kodak GelLogic 200 and the Carestream software. MW, GE Healthcare rainbow full range molecular weight marker.
(PDF)

S4 Fig Raw image used for the production of Fig 1C, 1D and 1E. Images were captured with the Kodak GelLogic 200 and the Carestream software. MW, GE Healthcare rainbow full range molecular weight marker.
(PDF)

S5 Fig Raw Image for used the production of S2 Fig. Image was captured with the Amersham Imager 680 MW, GE Healthcare rainbow full range molecular weight marker.
(PDF)
