# Correction: Evaluation of inactivated *Bordetella pertussis* as a delivery system for the immunization of mice with Pneumococcal Surface Antigen A

**DOI:** 10.1371/journal.pone.0229050

**Published:** 2020-02-05

**Authors:** Julia T. Castro, Giuliana S. Oliveira, Melissa A. Nishigasako, Anne-Sophie Debrie, Eliane N. Miyaji, Alessandra Soares-Schanoski, Milena A. Akamatsu, Camille Locht, Paulo L. Ho, Nathalie Mielcarek, Maria Leonor S. Oliveira

In the article title, the word “Antigen” should be “Protein.” The correct title is: Evaluation of inactivated *Bordetella pertussis* as a delivery system for the immunization of mice with Pneumococcal Surface Protein A. The correct citation is: Castro JT, Oliveira GS, Nishigasako MA, Debrie A-S, Miyaji EN, Soares-Schanoski A, et al. (2020) Evaluation of inactivated *Bordetella pertussis* as a delivery system for the immunization of mice with Pneumococcal Surface Protein A. PLoS ONE 15(1): e0228055. https://doi.org/10.1371/journal.pone.0228055.

## References

[1] Castro JT, Oliveira GS, Nishigasako MA, Debrie A-S, Miyaji EN, Soares-Schanoski A, et al (2020) Evaluation of inactivated *Bordetella pertussis* as a delivery system for the immunization of mice with Pneumococcal Surface Antigen A. PLoS ONE 15(1): e0228055 10.1371/journal.pone.0228055 31945121 PMC6964896

