# Supplementary figures and images for: Evaluation of inactivated Bordetella pertussis as a delivery system for the immunization of mice with Pneumococcal Surface Antigen A
Source: PLoS One. 2020 Jan 16;15(1):e0228055. doi: 10.1371/journal.pone.0228055 (PMC6964896; doi:10.1371/journal.pone.0228055)

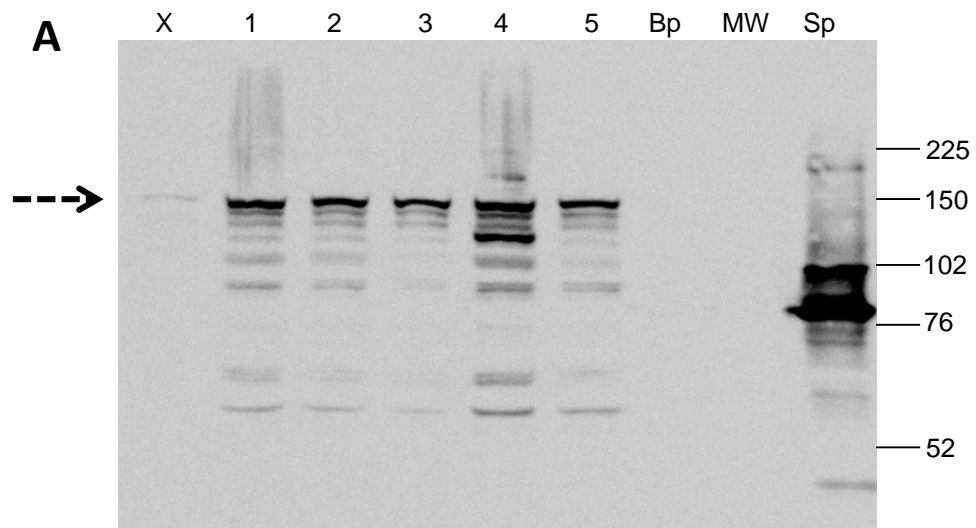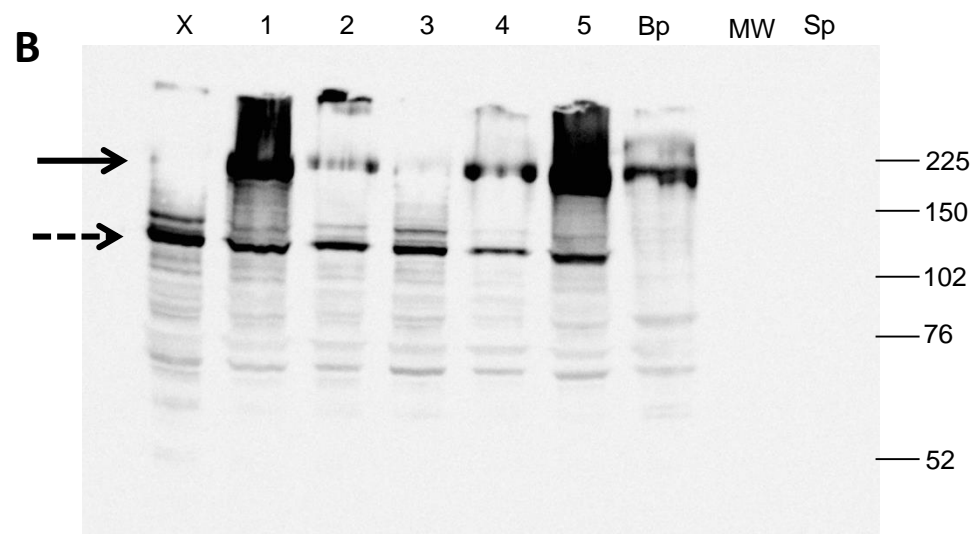

Supplement: S3 Fig — Images were captured with the Kodak GelLogic 200 and the Carestream software. MW, GE Healthcare rainbow full range molecular weight marker. (PDF) [file pone.0228055.s003.pdf]

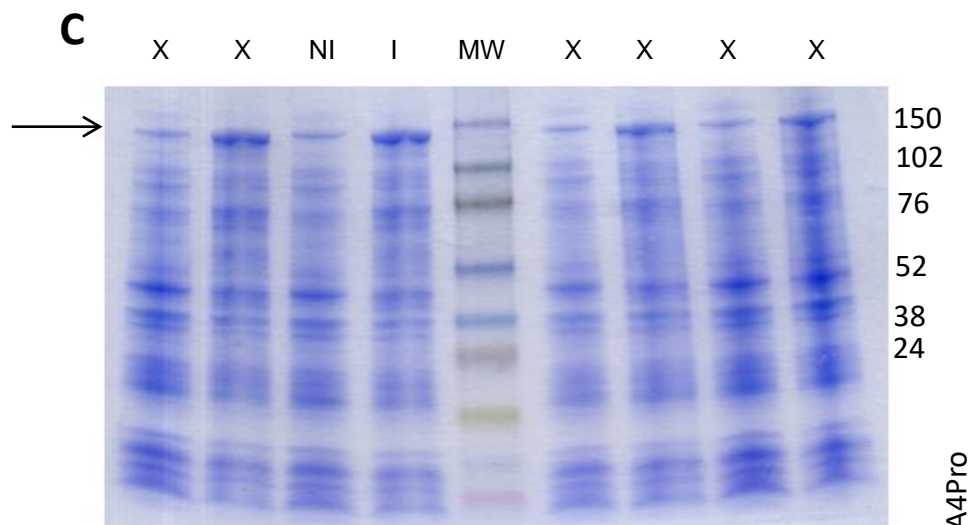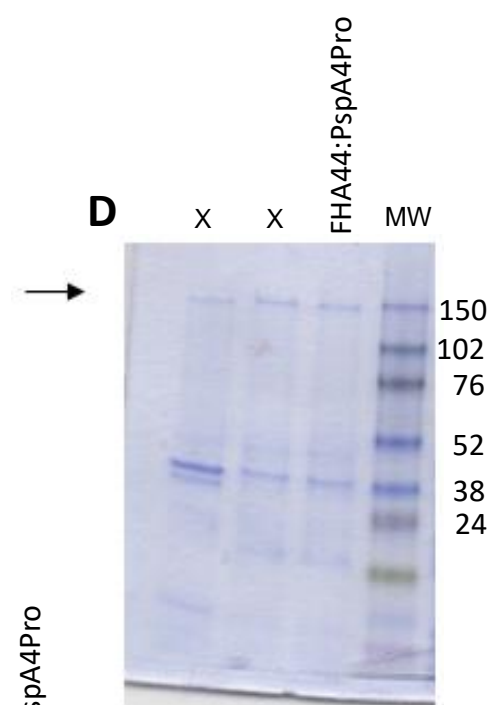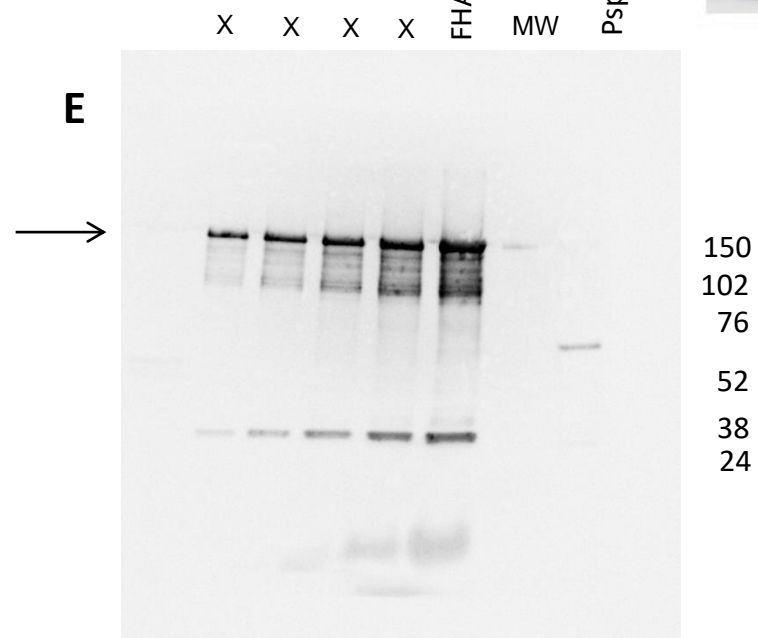

Supplement: S4 Fig — Images were captured with the Kodak GelLogic 200 and the Carestream software. MW, GE Healthcare rainbow full range molecular weight marker. (PDF) [file pone.0228055.s004.pdf]

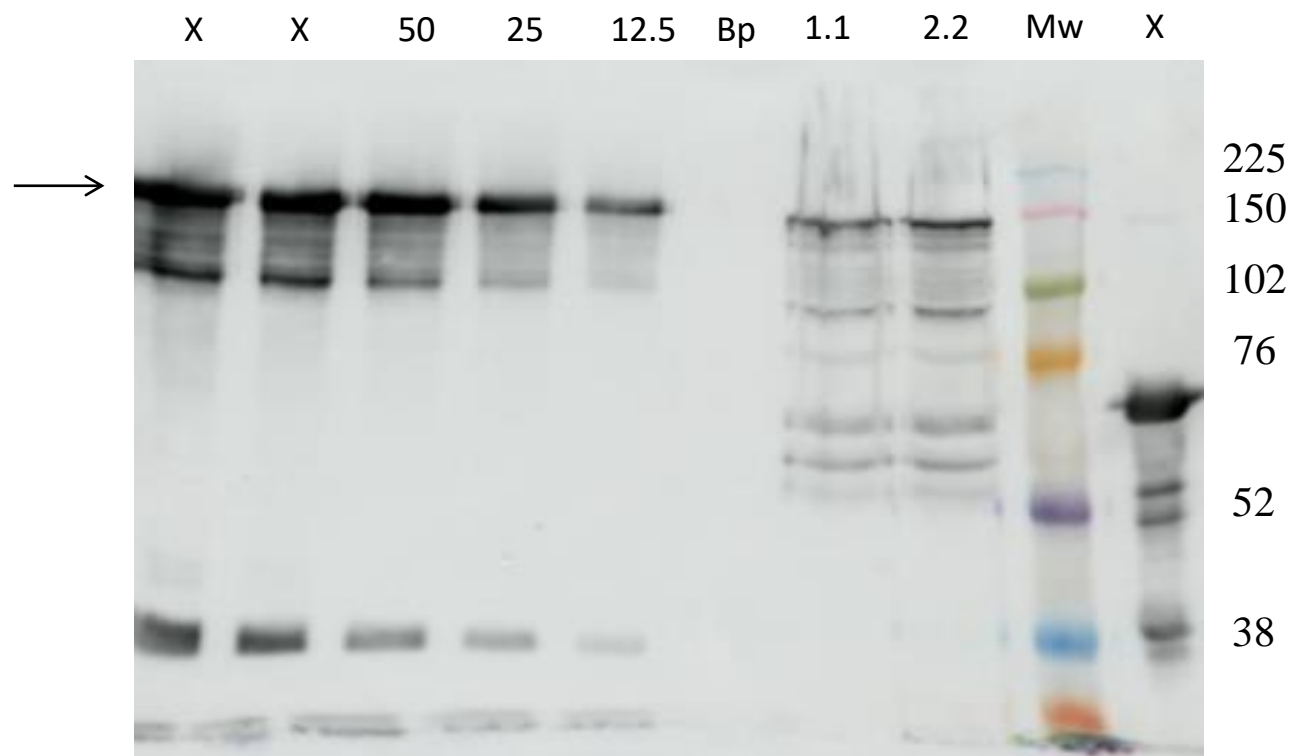

Supplement: S5 Fig — Image was captured with the Amersham Imager 680 MW, GE Healthcare rainbow full range molecular weight marker. (PDF) [file pone.0228055.s005.pdf]
